# How to Save Half a Million Dollars: An Antimicrobial Stewardship Program in a Tertiary Care Center

**DOI:** 10.7759/cureus.5121

**Published:** 2019-07-10

**Authors:** Balram Rathish, Arun Wilson, Anup Warrier, Rachana Babu, Shilpa Prakash

**Affiliations:** 1 Internal Medicine, Aster Medcity, Kochi, IND; 2 Infectious Diseases, Aster Medcity, Kochi, IND; 3 Clinical Microbiology, Aster Medcity, Kochi, IND; 4 Clinical Pharmacology, Aster Medcity, Kochi, IND

**Keywords:** antimicrobial stewardship, quality improvement, cost-effective

## Abstract

We started a quality improvement project (QIP) with the aim of implementing an antimicrobial stewardship program (AMSP) to optimize antimicrobial use. We implemented this QIP in our tertiary care center with baseline data from July 1, 2017, to December 31, 2017 (pre-AMSP period), and the project period between January 1, 2018, and June 30, 2018. It covered every inpatient with a positive microbiological culture and patients who were initiated on a pre-selected list of 16 restricted antimicrobials. Numerous plan-do-study-act (PDSA) cycles were conducted alongside daily AMSP rounds, consisting of prospective audit and feedback to all stakeholders. The outcome measures used were antibiotic consumption and costs, Clostridium difficile infection (CDI) rates, the average length of stay (LOS), and adverse drug reaction (ADR) reporting rates. We demonstrated a considerable reduction in the consumption of the selected antibiotics, as well as a direct translation to cost-saving. The AMSP directly contributed to collective savings of around half a million US dollars in hospital bills for patients. We also demonstrated reduced average LOS, CDI rates, and increased reporting of ADRs to antibiotics. The reduction in average LOS was also directly beneficial to patients with reduced time spent in the hospital. The reduction in CDI rates proves that there is a reduction in the development of AMR, and in the short term, fewer incidences of healthcare-associated infections. This, in addition to better surveillance of ADRs to antimicrobials, improved patient safety and quality of care.

## Introduction

The British Medical Journal's collection of 15 articles on "Antimicrobial Resistance in South East Asia,” highlighted the issue of antimicrobial resistance (AMR) in South Asia and showed that it has become a critical political, social, and economic problem in the World Health Organization (WHO) South-East Asia region, where there is possibly the highest risk globally for the emergence and spread of AMR [1]. The rampant use of antimicrobials in the medical, veterinary, and commercial industries have been postulated and proven to be the major causes for the development of AMR. We wanted to start a quality improvement project (QIP) with the aim of implementing an antimicrobial stewardship program (AMSP) to optimize antimicrobial use in our tertiary care center. Our challenge was to come up with such a program in a limited resource setting while maintaining the highest quality of care for our patients, without incurring any further expenses for the patients. The uniqueness of our AMSP has also been the focus on a prospective audit with daily feedback, rather than a formulatory restriction, with a consensual decision-making process involving the primary treating team and the AMSP team, with the key stakeholder being the patient, by ensuring the interests of the key stakeholder at the center of the process.

## Materials and methods

We implemented an AMSP as a QIP in our 430-bed private tertiary care center in Kochi, India, on January 1, 2018. A comparison of various measures was drawn between two six-month periods including baseline data, which was collected from July 1, 2017, to December 31, 2017 (designated as the pre-AMSP period), and the project period data that was collected between January 1, 2018, to June 30, 2018 (designated as the AMSP period).

The AMSP covered every inpatient who fit into the criteria for undergoing AMSP, which included having a positive microbiological culture and patients who were initiated on a pre-selected list of 16 restricted antimicrobials, including colistin, polymixin B, fosfomycin, daptomycin, teicoplanin, tigecycline, minocycline, meropenem, doripenem, ertapenem, imipenem, IV vancomycin, oral vancomycin, IV linezolid, oral linezolid, and flucloxacillin. The AMSP team consisted of an infectious diseases (ID) physician, clinical microbiologist, internal medicine trainee, as well as a clinical pharmacist trained in ID. Daily AMSP rounds were initiated at our center, where each patient who fit into the criteria was visited, the antimicrobial prescribed was reviewed, the clinical condition assessed, and the patient followed up until discharge. The appropriateness of the antimicrobial being used in the patient was decided based on the microbiological sample reports, the clinical syndrome, and its standard of care, as well as the discretion of the ID physician. Numerous PDSA cycles were conducted alongside daily AMSP rounds, comprising prospective audit and feedback to all stakeholders, including the primary treating physician, the patient and his/her bystander, and any other staff involved in the treatment of that particular patient.

The outcome measures used to measure the success of the AMSP were the consumption of the pre-selected 16 antibiotics and the costs incurred due to the prescription of these agents, Clostridium difficile infection (CDI) rates, average length of stay (LOS) of patients, and reporting rates of adverse drug reaction (ADR) to antibiotics. We measured the total number of doses of each of these antimicrobials that were prescribed to our inpatients during the pre-AMSP period and compared it to the AMSP period. The number of doses adjusted to inpatient days (IPD) during each of these two respective six-month periods were calculated as well. We also looked at the cost of each of these agents, and the overall expense incurred from the use of these 16 agents in the pre-AMSP as well as the AMSP period.

## Results

**Figure 1:**
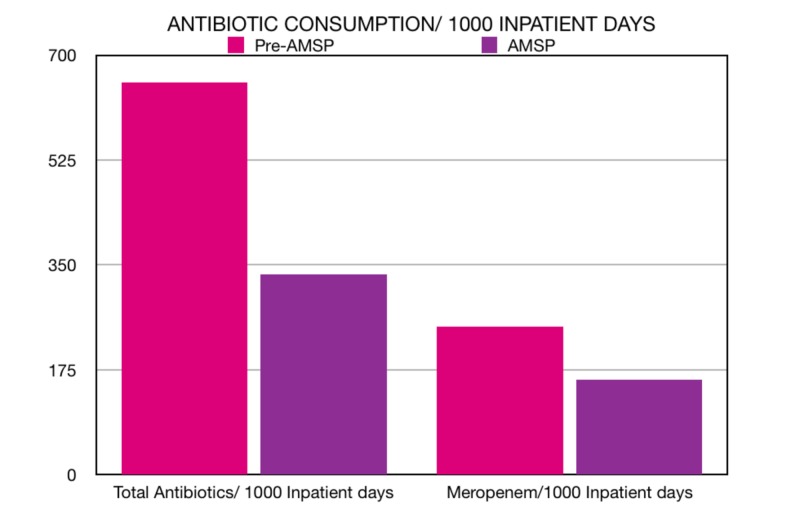
Total consumption of antibiotic doses per 1000 IPD in the pre-AMS period versus the AMS period (left) and consumption of meropenem doses per 1000 IPD in the pre-AMS period versus the AMS period (right). AMS: Antimicrobial Stewardship; IPD: Inpatient Days

We calculated the total number of doses of these pre-selected 16 agents that was administered to patients during the AMSP period (January 2018 to June 2018) and found it to be 247 doses/1000 IPD. This was compared to the total number of doses of these same 16 agents in the pre-AMSP period (July 2017 to December 2017), which was 654 doses/1000 IPD. Since the most widely used drug in this list of 16 agents was meropenem, we also compared the number of doses of meropenem that was administered during the pre-AMSP and AMSP periods. The use of meropenem reduced drastically from 334 doses per 1000 IPD during the pre-AMSP period to 159 doses per 1000 IPD during the AMSP period (Figure 1).

**Figure 2:**
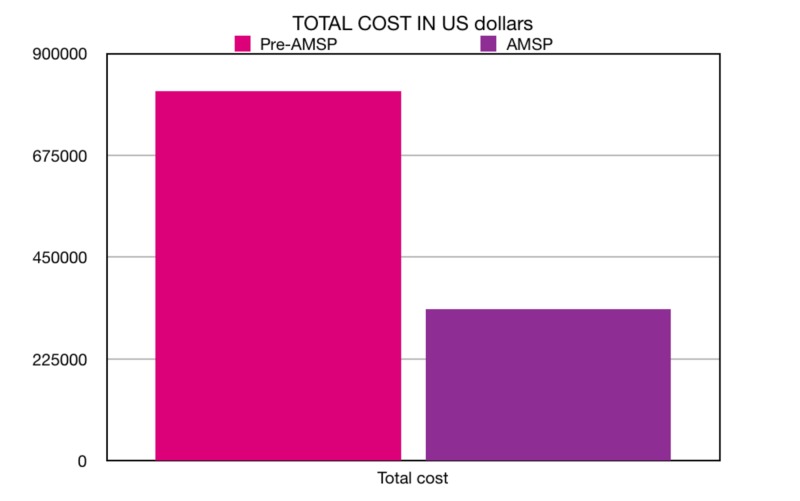
Total cost of antibiotics (in US dollars) consumed in the pre-AMS period versus the AMS period. AMS: Antimicrobial Stewardship

Similarly, the average cost of these 16 agents during the AMSP period was calculated to be 2,38,94,526 Indian rupees (INR) or 336,069 US dollars in comparison to the pre-AMSP period of 5,72,67,079 INR or 816,934 US dollars. The net difference our AMSP made was a reduction of 407 doses of these selected agents per 1000 IPD, which directly translated into costs as well, where we were able to save nearly half a million US dollars (Figure 2).

**Figure 3:**
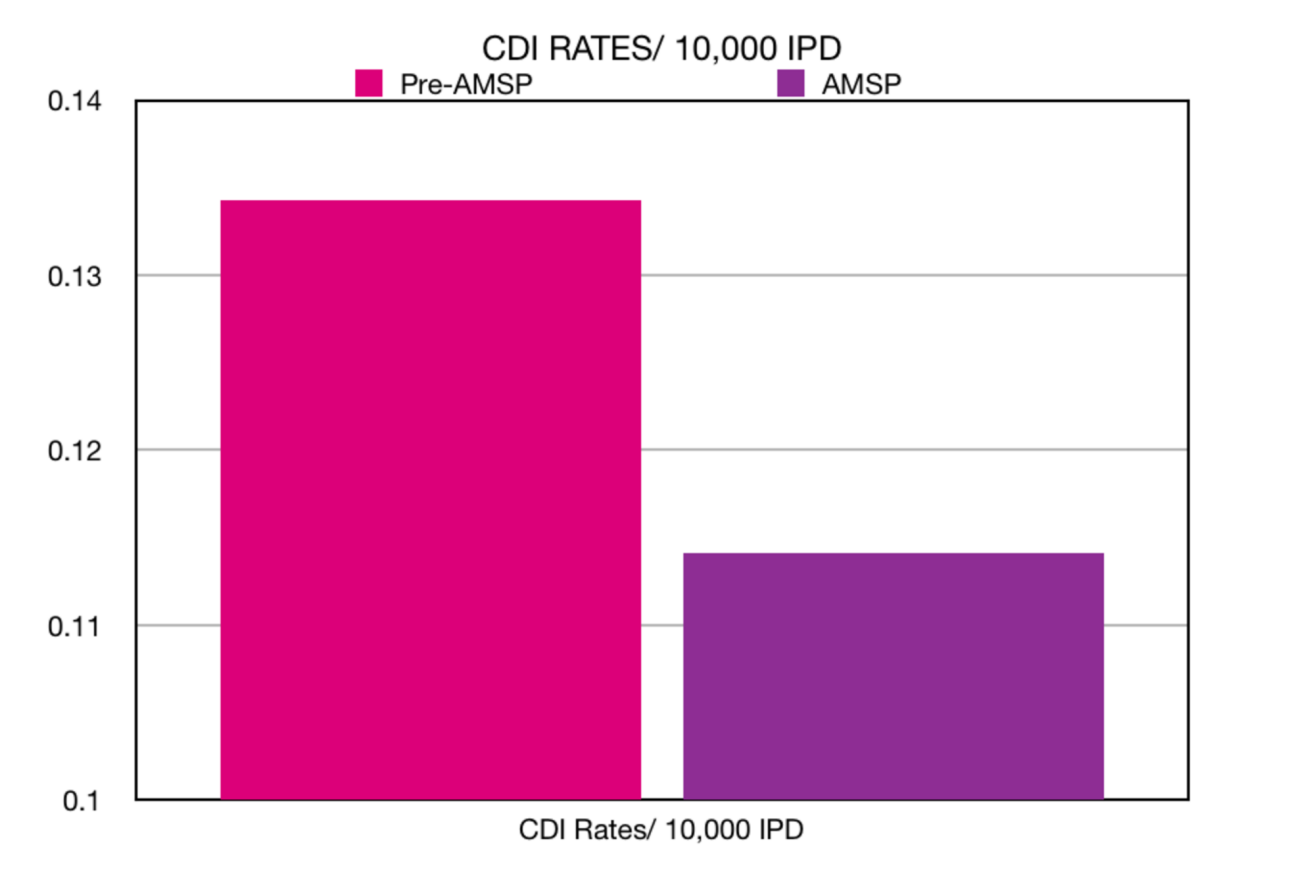
Clostridium difficile infection (CDI) rates/10,000 inpatient days in the pre-AMS period versus the AMS period. AMS: Antimicrobial Stewardship; CDI: Clostridium Difficile Infection; IPD: Inpatient Days

The Clostridium difficile infections/inpatient days (CDI/IPD) rates were calculated to be 0.1141/10,000 inpatient days during the AMSP period in comparison to the pre-AMSP CDI/IPD rates of 0.1343/10,000 inpatient days. We demonstrated a reduction of 0.0202/10,000 patient days in Clostridium difficile infections (Figure 3).

**Figure 4:**
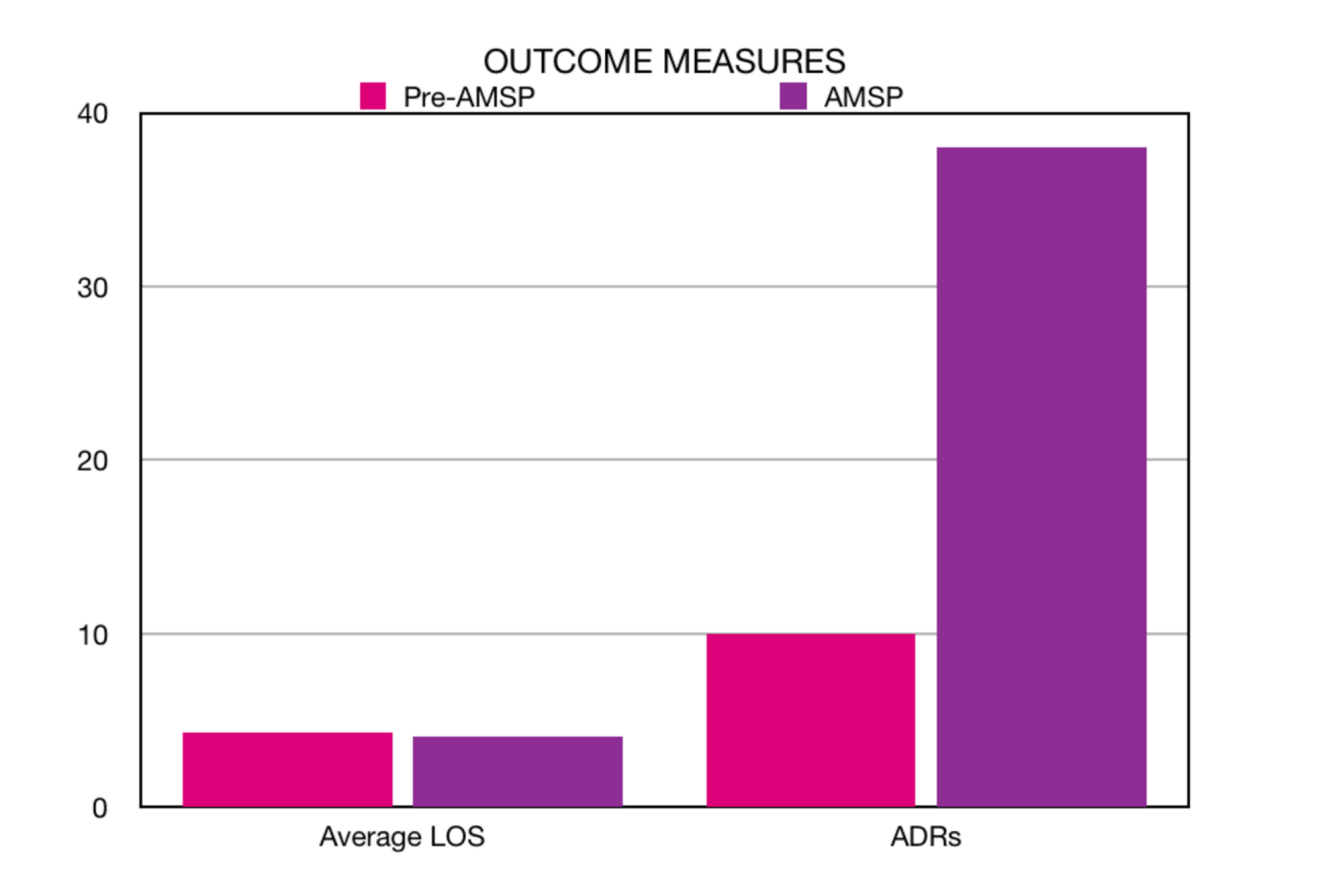
Average length of stay (LOS) in days in the pre-AMS period versus the AMS period (left) and the number of ADRs reported in the pre-AMS period versus the AMS period (right). AMS: Antimicrobial Stewardship; LOS: Length of Stay; ADR: Adverse Drug Reaction

Our next clinical outcome was the average LOS, which is an indirect marker of a successful AMSP and patient morbidity. The AMSP period average LOS was measured to be 4.13 days/patient against the pre-AMSP average LOS of 4.3 days/patient. There was a reduction of 0.17 days/patient in the AMSP period (Figure 4).

A process measure we decided to look at was the number of reportings of ADRs to antimicrobials. Prior to the initiation of the AMSP, there was only a passive system for the reporting of ADRs to antimicrobials, and this was being done by any member of the primary treating team. There was no dedicated personnel to monitor ADRs. During the AMSP period, our clinical pharmacist was designated to record all ADRS to antimicrobials and we found the number to be 38 ADRs in six months, averaging 6.33 ADRS per month in contrast to the pre-AMSP period ADRs of 10 in six months, averaging 1.66 per month, where there was obvious under-reporting due to the absence of dedicated personnel for documenting ADRs to antimicrobials (Figure 4).

## Discussion

The first article using the term ‘Antimicrobial Stewardship’ was published by McGowan et al. in 1996; they wanted to highlight that we should consider antimicrobials a precious non-renewable resource, using a term that incorporated both the appropriate use of antimicrobials when they are indicated, as well as avoid unnecessary use [2]. The highlight among the outcome measures in our AMSP QIP was the extent of reduction in antibiotic use and the cost involved. We could demonstrate a considerable reduction in the usage of our selected antibiotics, and our project saved our patients around half a million US dollars in a mere six months with a team consisting of only four members. This was achieved by the model of a QIP, with daily PDSA cycles consisting of prospective feedback to all relevant stakeholders in direct contrast to commonly employed AMSP methods, such as the formulatory restriction of antibiotics, which can lead to reduced compliance by potential limitations, including delays in therapy, prescriber pushback, and unintended increases in the use of unrestricted antimicrobials [3].

The indirect impact of our AMSP results in even bigger savings. A reduction in the average length of stay of our patients translates to reduced hospital bills. This is also true of reduced Clostridium difficile infections, which indicates reduced healthcare-associated infection rates and subsequent expenditure for the treatment of the same. Better surveillance of adverse drug reactions (ADR) to antimicrobials also translated directly to cost-savings by the early identification and management of these ADRs, with a prompt change in the agent, to prevent more such episodes and, most importantly, improve patient safety and quality of care.

## Conclusions

An antimicrobial stewardship program may improve patient outcomes and can significantly reduce healthcare-related expenditure. Following the quality improvement (QI) model of regular prospective feedback to relevant stakeholders proved to be an effective method, even with a relatively small AMSP team, which enables this model to be easily reproduced in resource-limited settings like India where there may be a dearth of manpower as well as the availability of antibiotics. The QI model also proved to provide significant gains in terms of healthcare expenditure in a relatively short period of time, which further adds validation over more traditional models of AMSP.
